# Variations in Lead Isotopic Abundances in Sprague-Dawley Rat Tissues: Possible Reason of Formation

**DOI:** 10.1371/journal.pone.0089805

**Published:** 2014-02-25

**Authors:** Duojian Liu, Jing Wu, Li Ouyang, Jingyu Wang

**Affiliations:** 1 School of Public Health, Peking University, Haidian District, Beijing, People’s Republic of China; 2 Center of Medical and Health Analysis, Peking University, Beijing, People’s Republic of China; University of Kentucky, United States of America

## Abstract

It has been reported in previous research that the lead isotopic composition of blood, urine and feces samples statistically differed from the given lead sources in Sprague-Dawley (SD) rats. However, the reason for this phenomenon is still unclear. An animal experiment was performed to investigate the lead isotope fractionation in diverse biological samples (i.e., lungs, liver, kidneys, bone) and to explore the possible reasons. SD rats were intratracheally instilled with lead acetate at the concentrations of 0, 0.02, 0.2, and 2 mg/kg body weight. Biological samples were collected for lead isotope analysis using an inductively coupled plasma mass spectrometry (ICP-MS). Significant differences are observed in lead isotope abundances among the diverse biological samples. The lead isotope abundances (^206^Pb, ^207^Pb and ^208^Pb) in diverse biological samples show different degrees and directions of departure from the given lead source. The results suggest that differences in enrichment or depletion capacity for each lead isotope in the various tissues might lead to the variation in lead isotopic abundances in tissues. Moreover, a nonlinear relationship between the blood lead level and the lead isotope abundances in liver and bone is observed. When the whole-blood level is higher than 50 ng/mL, the lead isotopic compositions of biological samples tend to be the same. Thus, the data support the speculation of a fractionation functional threshold.

## Introduction

Lead (Pb) poisoning is a long-standing and serious environmental problem for living organisms and can induce adverse health effects [1]–[2]. According to the recent scientific literature, metal smelters, battery recycling, electronics, paint, traditional remedies and leaded gasoline are primary sources of lead exposure [3]. A key strategy for reducing human health risk of lead poisoning is to identify and control the lead sources.

There are four naturally occurring stable isotopes of lead (^204^Pb, ^206^Pb,^207^Pb and ^208^Pb), three of which are radiogenic and produced by the radioactive decay of uranium and thorium [4]. The fourth isotope of lead (^204^Pb) is non-radiogenic. The isotopic composition of lead from any geologic sources was fixed when the ore was formed [5]. Thus, each geologic source has a characteristic isotopic “signature” consisting of variable abundances of four stable isotopes. Due to its small fractional mass differences, it is generally assumed that lead isotopes do not fractionate measurably in biological systems [6]–[7], and can be utilized as a tracer of Pb provenance in a variety of complex environments [4], [8]–[9].

However, Smith reported that there were large differences in the lead isotopic composition between paired blood and bone samples from each human subject [10]. In addition, distinct differences in ^206^Pb/^208^Pb ratio were found among kidneys, liver and other tissues in rats fed with a single enriched lead isotope source [11]. Our previous approach showed a similar phenomenon that there were remarkable differences in lead isotopic signature among the biological samples [12], suggesting that there might be a biological fractionation of lead isotopes in different biological samples.

Regardless of which route entry into the body, exogenous lead binds to erythrocytes and travels in the bloodstream to soft tissues, such as liver, kidneys, lungs and heart, and excretes into urine, feces, sweat and so on [13]. In order to further investigate the phenomenon of biological fractionation in vivo, it is necessary to examine the lead isotopic composition of different tissues which are important in the metabolism processes, such as kidneys, liver, lungs and bone. To date, there are few publications pertaining to lead isotopic fractionation in living creature or why biological fractionation may occur. The lead isotope analysis of tissues may contribute to the understanding of the lead isotope distribution in tissues and provide clues to the reason for lead isotopic fractionation, and provide some help to determine the suitable biological marker of identifying lead pollutant sources.

Therefore, an animal experiment was carried out with the following aims: 1) to investigate whether the lead isotope fractionation occurs in tissues of rats, 2) to explore the possible reason for biological fractionation of lead isotopes, and 3) to verify whether the threshold of biological fractionation function would be observed in tissues of rats.

## Materials and Methods

### Animal experiments

Six-week-old male S-D rats (210–250 g) were sourced from the Department of Laboratory Animal Science, Peking University Health Science Center. Rats were housed in plastic cages with free access to food and water, and maintained in a constant room with controlled temperature, relative air humidity and 12-hour light and dark circles. All animals protocols were approved by Peking University Health Science Center's Ethics Committee on Animal Experiment (Permit Number: LA2012-60).

After a three-day acclimation period, 24 rats were randomly assigned to four groups (6 animals/group), including the control group and three experimental groups. Control rats received 0.1 mL deionized water intratracheally. The rats in experimental groups (low-dose, medium-dose and high-dose groups) were intratracheally instilled with 0.02, 0.2 and 2 mg/kg body weight of lead acetate (namely test substance), respectively. Lead acetate solutions of different concentrations were prepared from lead acetate trihydrate. The animals were treated in this way every day for 5 days.

Twenty-four hours after the last administration, the rats were anesthetized with ether and sacrificed. At the time of necropsy, the organs (including lungs, livers, kidneys, and femurs) were taken from each individual. After trimming of extraneous fat and washing by deionized water, the organs were placed in polyethylene bags and stored at –20°C until analyses. We also collected the lead acetate, food, water and air (the total suspended particles collected by an air sampler) samples that the rats get access to.

### Sample preparation

**Reagents.** Nitric acids (UP), perchloric acid (GR), ammonium hydroxide (UP), lead acetate trihydrate (AR), and anhydrous diethyl ether were utilized in this study. Deionized water (18 MΩ cm) prepared by de-ionization of reverse osmosis water using GN-RO-100 purification system, was used to prepare all solutions. To prevent contamination, all labware was immersed in ∼50% HNO_3_ solution for at least 12 hours, and washed 20 times with deionized water.

The lead isotopic compositions of the diet and the test substances were both constant throughout the study, but significant differences between these lead sources exist in ^204^Pb, ^207^Pb and ^208^Pb abundance (Table 1).

**Table 1:** Four lead isotope abundances (mean ± SD) for diet and test substance.

Lead source	^204^Pb%	^206^Pb%	^207^Pb%	^208^Pb%
Diet	1.364±0.007^a^	24.952±0.052	21.249±0.044^a^	52.438±0.045^a^
Test substance	1.373±0.001	24.902±0.027	21.396±0.026	52.335±0.047

a significant difference between diet and test substance at *P*<0.05.

**Sample digestion.** For the organ samples, about 1.0 g (except femurs 0.2 g) were weighed out into quartz vials. The samples were digested in two steps: 1) leached with proper volume mixed acid (HNO_3_ and HClO_4_ at 20:1volume ratio) under ambient conditions for at least 12 hours, and 2) heating on a hotplate until the digested sample solution became clear and transparent. The digestion residue was transferred to centrifuge tubes with deionized water. To reduce the matrix effect, the digested sample solution for analysis followed the procedures of enrichment and separation of Pb described in Wu et al [14]. All samples solutions were prepared freshly for Pb measurement session by diluting with 1% HNO_3_ solution.

### Analysis by ICP-MS

**Standards.** Pb standard solutions used for concentration determination were prepared daily from 1000 µg/mL Pb solution from National Research Center for Certified Reference Materials. The SRM 981 Pb isotopic standard (National Institute of Standards and Technology, NIST, USA) was applied for normalization of measured Pb isotopic abundance and monitoring analytical run instrument drift.

**Analytical techniques.** Lead concentrations and isotope analyses were performed on a Perkin-Elmer ICP-MS Elan DRCII, which is housed in a trace metal-clean HEPA filtered air (Class 1000) lab at Medical and Pharmaceutical Analysis Center, Peking University. Lead measurements were obtained following the same operating parameters for ICP-MS in our previous study [14]. The isobaric Hg interference on ^204^Pb was corrected by monitoring ^202^ Hg signal.

At the start of each analytical session a batch (n = 6) of the SRM 981 standard was run. Samples were run following a standard samples-standard measurement protocol, where the standard was run after every eight samples. The SRM 981 isotopic abundance was used for the correction factors of calculation. In this study we calculated the relative standard deviation (RSD) of the batch of 10 ng/ml SRM 981 solution, the RSD values for the ^204^Pb,^ 206^Pb,^207^Pb,^208^Pb abundance is 0.124%, 0.054%, 0.084%, 0.027%, respectively.

### Statistical treatment of the data

SPSS software (Version 16.0, SPSS Inc.) was used for all statistical analyses. One-way analysis of variance(ANOVA)test was used for comparison of isotopic abundance among different groups or samples. The heterogeneous cases were analyzed with Kruskal –Wallis rank sum test. The isotopic abundance comparison between biological samples and test substance (diet) was analyzed with the independent samples t-test. The nonparametric analysis was used when the data were not normally distributed. A *p* value of <0.05 was considered statistically significant.

## Results

### Comparison of lead isotope abundances between biological samples-diet and biological samples-test substance pairs

For the ^204^Pb abundance, significant differences were found between the kidney-test substances pairs in low- and high-dose groups. The ^204^Pb abundance of urine and liver differed from that of test substance in medium-dose group and high-dose group, respectively (Table 2). In the case of ^206^Pb abundance, significant differences were observed between all biological samples-test substance pairs in medium- and high-dose group, and the blood, urine, kidneys and lungs samples were significantly different from the test substance in low-dose group (Table 3). The ^207^Pb abundance showed significant differences between the urine-test substance pairs in experimental groups, while no significant difference was observed between the blood, feces and kidneys samples-test substance pairs (Table 4). In the control group, the blood, urine, lungs and bone samples differed from the diet for ^206^Pb and ^207^Pb abundances.

**Table 2:** ^204^Pb% (mean ± SD) for biological samples in different groups (n = 6).

Sample	Control	Low-dose	Medium-dose	High-dose
Blood	1.374±0.020	1.364±0.015	1.369±0.008	1.368±0.007
Urine	1.379±0.007^D^	1.371±0.009	1.383±0.009^D,T^	1.375±0.005^D^
Feces	1.366±0.005	1.369±0.005	1.373±0.004^D^	1.370±0.006
Kidney	1.370±0.010	1.367±0.005^T^	1.369±0.006	1.364±0.004^T^
Liver	1.383±0.035	1.364±0.010	1.368±0.010	1.368±0.003^T^
Lung	1.386±0.037	1.368±0.018	1.369±0.009	1.368±0.007
Bone	1.403±0.021^D^	1.374±0.036	1.378±0.019^g^	1.365±0.010^g,h^

Note: Difference are significant when *p<*0.05 level.

D,T A significant difference with diet and test substance, respectively.

g,h A significant difference with control group, low-dose group, respectively.

**Table 3:** ^206^Pb% (mean ± SD) for biological samples in different groups (n = 6).

Sample	Control	Low-dose	Medium-dose	High-dose
Blood	24.512±0.086^D^	24.632±0.054^D,T,g^	24.714±0.057^D,T,g,h^	24.783±0.043^D,T,g,h^
Urine	24.636±0.063^D^	24.654±0.033^D,T^	24.693±0.065^D,T^	24.691±0.045^D,T,a^
Feces	24.965±0.108^a,b^	24.940±0.072^a,b^	24.831±0.034^D,T,a,b,g,h^	24.788±0.041^D,T,b,g,h^
Kidney	24.908±0.094^a,b^	24.801±0.045^D,T,a,b,c,g^	24.799±0.011^D,T,a,b,g^	24.771±0.020^D,T,b,g^
Liver	24.956±0.192^a,b^	24.916±0.122^a,b,d^	24.830±0.060^D,T,a,b^	24.778±0.022^D,T,b,g,h^
Lung	24.763±0.056^D,a,b,c,d,e^	24.756±0.038^D,T,a,b,c,e^	24.762±0.047^D,T,b,c,e^	24.809±0.029^D,T,b^
Bone	24.837±0.087^D,a,b,c^	24.798±0.101^D,a,b,c,e^	25.010±0.064^T,a,b,c,d,e,f,g,h^	24.822±0.027^D,T,b,d,e,i^

Note: Difference are significant when *p<*0.05 level.

D,T A significant difference with diet and test substance, respectively.

a,b,c,d,e,f A significant difference with blood, urine, feces,kidneys, liver and lungs, respectively.

g,h,i A significant difference with control group, low-dose group and medium-dose group, respectively.

**Table 4:** ^207^Pb% (mean ± SD) for biological samples in different groups (n = 6).

Sample	Control	Low-dose	Medium-dose	High-dose
Blood	21.520±0.081^D^	21.426±0.046^D,^ g	21.434±0.054^D,^ g	21.367±0.033^D,^ g
Urine	21.556±0.094^D^	21.507±0.064^D,T,a^	21.516±0.064^D,T,a^	21.481±0.065^D,T,a^
Feces	21.315±0.064^a,b^	21.374±0.055^D,b^	21.369±0.026^D,a,b^	21.390±0.011^D,b^
Kidney	21.320±0.091^a,b^	21.385±0.033^D,b^	21.379±0.020^D,a,b^	21.391±0.010^D,b^
Liver	21.343±0.157^a,b^	21.308±0.083^T,a,b^	21.344±0.020^D,T,a,b^	21.402±0.019^D,b^
Lung	21.389±0.075^D,a,b^	21.408±0.031^D,b,e^	21.433±0.019^D,T,b,c,d,e^	21.384±0.028^D,b^
Bone	21.459±0.095^D,c,d,e^	21.476±0.057^D,T,c,d,e,f^	21.444±0.052^D,b,c,d,e^	21.399±0.039^D,b^

Note: Difference are significant when *p<*0.05 level.

D,T A significant difference with diet and test substance, respectively.

a,b,c,d,e,f A significant difference with blood, urine, feces,kidneys, liver and lungs, respectively.

g A significant difference with control group.

When the ^208^Pb% was considered (Table 5), statistical differences between all biological sample-test substance pairs were found in experimental groups, except the feces and liver samples-test substance pairs in low-dose group. In control group, blood, feces and bone samples were statistically different from the diet.

**Table 5:** ^208^Pb% (mean ± SD) for biological samples in different groups (n = 6).

Sample	Control	Low-dose	Medium-dose	High-dose
Blood	52.596±0.065^D^	52.581±0.056^D,T^	52.486±0.040^T,g,h^	52.486±0.054^T,g,h^
Urine	52.430±0.072^a^	52.470±0.042^T,a^	52.410±0.058^T,a^	52.456±0.051^T^
Feces	52.355±0.053^D,a^	52.319±0.049^D,a,b^	52.431±0.054^T,g,h^	52.455±0.033^T,g,h^
Kidney	52.410±0.041^a^	52.456±0.038^T,a,c^	52.460±0.024^T^	52.480±0.026^T^
Liver	52.328±0.214^a^	52.421±0.073^T,a,c^	52.466±0.049^T^	52.458±0.038^T^
Lung	52.469±0.092^c^	52.475±0.049^T,a,c^	52.442±0.044^T^	52.446±0.019^T^
Bone	52.309±0.077^D,a,b,f^	52.360±0.134^a,b,d,f^	52.181±0.061^D,T,a,b,c,d,e,f,g,h^	52.421±0.043^T,i^

Note: Difference are significant when *p<*0.05 level.

D,T A significant difference with diet and test substance, respectively.

a,b,c,d,e,f A significant difference with blood, urine, feces,kidneys, liver and lungs, respectively.

g,h,i A significant difference with control group, low-dose group and medium-dose group, respectively.

### Comparison of lead isotope abundances among biological samples and among different groups

There was no statistical difference among biological samples in any groups for ^204^Pb%.When ^206^Pb% was considered, blood and urine samples were statistically different from all other biological samples in control, low-dose and medium-dose group, while the blood-lungs pair was indistinguishable in medium-dose group. Significant differences were found between bone-other sample pairs in medium-dose group, as well as urine-other samples pairs in high-dose group.

For ^207^Pb%, statistical differences were observed between all the pairs of bone-feces, bone-kidneys, bone-liver and blood-liver in each group except the high-dose group.The ^207^Pb abundance of urine sample was statistically different from feces, kidneys, liver, lungs samples in control and low-dose group, and from other samples in medium- and high-dose groups.

For ^208^Pb%, remarkable differences were observed between blood-other biological samples pairs in control and low-dose groups except the blood-lungs pair in the control group. The ^208^Pb abundance of bone differed from other biological samples in medium-dose group, and that of all biological samples were indistinguishable in high-dose group.

The comparison of lead isotopic abundance among different dose groups was shown in Tables 2–5. For urine and lungs samples, no significant difference was observed between any two groups for any isotopic abundance. Thus, the isotopic composition of urine and lungs were relatively constant.

### The isotopic abundance relationship among biological samples

**Figure 1:**
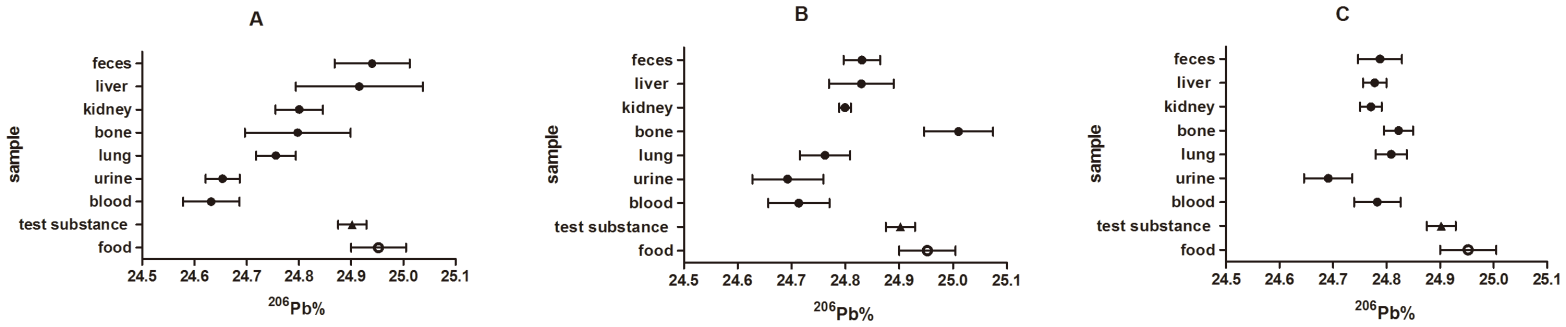
The ^206^Pb abundance of biological samples of S-D rats in relation to the ^206^Pb abundance of diet and test substance. Values indicated are means (n = 6) ± standard deviations. A, B, C represents the low-dose group, medium-dose group and high-dose group, respectively.

**Figure 2:**
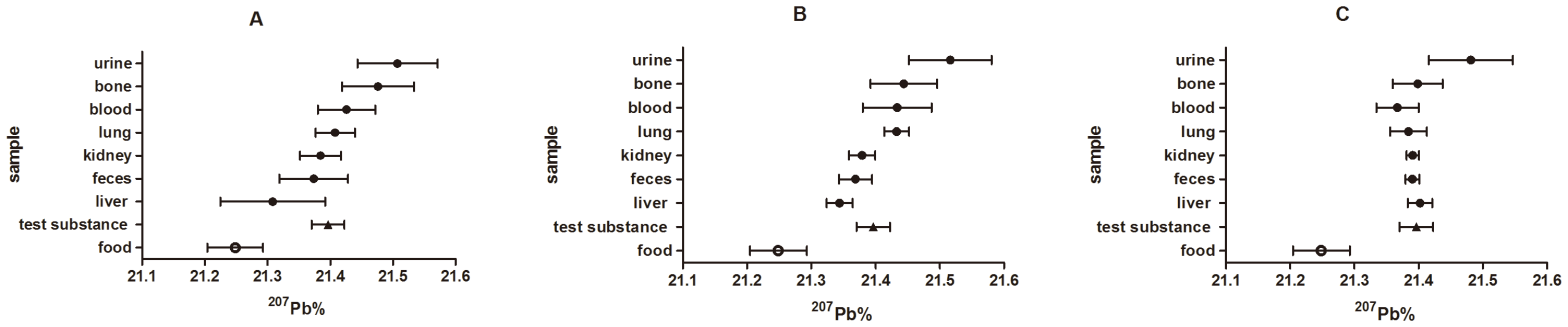
The ^207^Pb abundance of biological samples of S-D rats in relation to the ^207^Pb abundance of diet and test substance. Values indicated are means (n = 6) ±standard deviations. A, B, C represents the low-dose group, medium-dose group and high-dose group, respectively.

**Figure 3:**
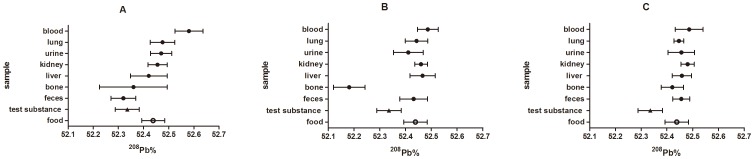
The ^208^Pb abundance of biological samples of S-D rats in relation to the ^208^Pb abundance of diet and test substance. Values indicated are means (n = 6) ±standard deviations. A, B, C represents the low-dose group, medium-dose group and high-dose group, respectively.

Three figures were utilized to illustrate the relationship among biological samples for ^206^Pb,^ 207^Pb and ^208^Pb abundances (Fig.1–3). For ^207^Pb abundance, the general relationship among biological samples (i.e. ^207^Pb% value, liver < feces < kidneys < lungs < blood < bone < urine) were observed in low-dose and medium-dose groups (Fig.2). In the low-dose group, the ^206^Pb% values were ranked blood < urine < lungs < bone < kidneys < liver < feces, and the ^208^Pb% values were ranked feces < bone < liver < kidney < urine, lung < blood. In medium-dose group, the rank trend for ^206^Pb abundance except bone generally remained the same, while the relationship for ^208^Pb abundance changed. Compared to the other samples, the ^206^Pb and ^208^Pb abundances in bone exhibited the opposite departure from the test substances in the medium-dose group.

The rank trend in lead isotopic abundances among biological samples altered or nearly disappeared in high-dose group. In general, most biological samples were depleted in ^206^Pb and enriched in ^208^Pb compared to the test substance in the high-dose group, in which the ^207^Pb abundance of test substance was nearly close to that of all biological samples except urine.

### The relationships between Pb isotopic abundances in biological samples and blood lead concentration

**Figure 4:**
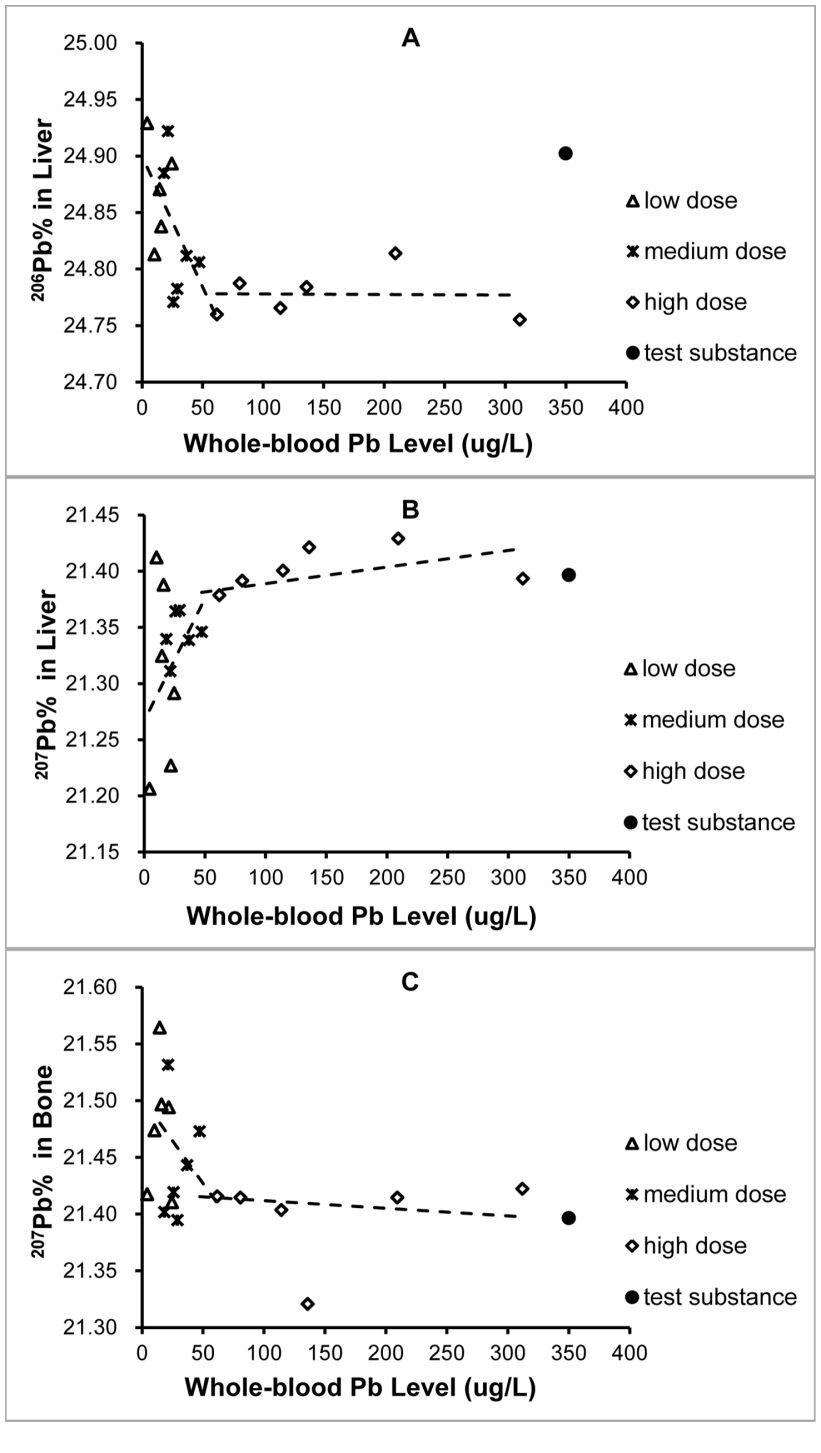
Whole-blood lead concentration versus lead isotope abundances in tissues for experimental groups (n  =  18). A. ^206^Pb% in liver. B. ^207^Pb% in liver. C. ^207^Pb% in bone.

In this study, the ^206^Pb% in liver and the ^207^Pb% in bone both were negatively associated with whole-blood lead level, while the ^207^Pb% in liver were positively associated with whole-blood lead level (Figure 4). Take Figure 4A for example, the ^206^Pb% in liver decreased with increasing whole-blood lead concentration and then reached a plateau. An inflection point could be seen for the lead isotopic abundance vs. the whole-blood lead level. A similar situation was observed in ^207^Pb% in bone vs. the whole-blood lead level. The ^207^Pb% in liver increased with increasing whole-blood lead concentration and then reached a plateau.

## Discussion

It has been generally recognized that the range in natural isotopic variation will decrease with increasing atomic number because the relative mass difference also decreases [15]. The element isotopic variation with larger range is more likely to be measured when the accuracy and precision of isotope determination is limited. As such, isotope fractionation for light elements, such as Li, C and N, has been known to occur in biological systems for several decades [16]–[18]. In recent years as analytical techniques have improved, isotopic variations for heavier elements (i.e. Zn, Fe and Hg) have also been observed in biological systems [19]–[21]. In view of the evidences, biological fractionation of lead isotopes is likely to occur as well.

As lead has four natural stable isotopes, there are six combinations of lead isotopic ratios in principle [22]. The geologists usually utilize the ratios ^206^Pb/^204^Pb, ^207^Pb/^204^Pb and ^208^Pb/^204^Pb which yield a relatively large variability between reservoirs [23].In environment provenancing studies, the isotopic composition of Pb is commonly presented as ratios^ 206^Pb/^207^Pb and ^208^Pb/^206^Pb [10], [24]. The ^206^Pb/^207^Pb ratio is the most preferred because it can be measured precisely analytically. However, the lead isotopic ratios used in environmental tracing research are not exactly the same [9], [25]. In fact, the isotopic composition of Pb can also be expressed as isotopic abundances, which not only carry the lead fingerprint information, but also are beneficial to represent the different degrees and directions of fractionation. Therefore, the isotopic abundances may be more suitable for the study aiming at investigating lead isotopic fractionation in the diverse samples.

In this study, lead isotope abundances of biological samples collected from lead poisoned SD rats were investigated. When the difference in isotopic composition between compared samples were greater than twice the measurement error, the two samples can be considered isotopically distinct [12].The data showed that the ranges of lead isotopic abundance between any two samples were 0.365–2.819%, 0.286–1.831%, 0.173–1.267%, 0.143–0.583% for ^204^Pb%,^ 206^Pb%, ^207^Pb% and ^208^Pb%, respectively. Therefore, differences in lead isotope abundances were larger than twice the corresponding RSD values (as described in analytical techniques). It can be concluded that differences in isotopic abundances between compared samples are reliable.

The data showed that lead isotope abundances in bone, kidneys, liver and lungs samples significantly differed from those in the given lead sources (Tables 2–5). In fact, our previous study has demonstrated that the lead isotopic compositions of blood, urine and feces samples were significantly distinguishable, and differed from those of given lead sources as well [12]. In order to investigate the lead isotopic variation during the absorption, distribution, metabolism and excretion processes in the body, it is necessary to compare the lead isotopic composition in blood, excretions (urine and feces), bone and soft tissues (lungs, kidneys, liver). The obtained data indicated that the lead isotope abundances had significant differences among seven biological samples from the same lead poisoned rats. These results are consistent with those of the Smith’ research, that there were discernible differences in lead isotopic composition between paired blood and bone samples from each human subject [10].

In this study, the data showed that the lead isotopic abundances of biological samples increased orderly (Figure 1–3). For example, the ^207^Pb% values were ranked liver < feces < kidneys < lungs < blood < bone < urine in low- and medium-dose groups. However, the rank relationship for lead isotopic abundances among biological samples nearly disappeared in the high-dose group. Another Pb isotopic approach described that the ^207^Pb/^206^Pb and ^208^Pb/^206^Pb ratios in vegetables increased in the order of roots < stems < leaves < fruits [26]. In addition, it has been already observed that the isotopic compositions of elements in different biological samples exhibited an increasing trend [17], [27]–[28], such as the δ^30^Si values for different organs of rice (stem < root < leaf < husk <grain) and the δ^13^C values for different tissues of gerbil (fat < liver < muscle < brain < hair).

According to the figures, the biological samples showed different degrees and directions of departure from the test substance. The degree of deviation in lead isotope abundances between the sample and the test substance reflect the enrichment or depletion capacity of the given bio-sample. Most biological samples were depleted in ^206^Pb relative to the test substance in experimental groups. The blood and urine samples had the greatest depletion and differed statistically from all other samples. By contrast, the majority of biological samples were enriched in ^208^Pb over the test substance. Blood showed the largest departure from the respiratory ^208^Pb in any experimental group. In the case of ^207^Pb, urine showed the greatest enrichment in experimental groups, while liver had greater depletion than any other samples in low-dose and medium-dose groups. Thus, the results indicated that the diverse tissues in rats possess different enrichment or depletion capacity for each given lead isotope.

Miller observed significant differences in lead isotopic compositions between the bone and fleshy tissues of rainbow trout [29]. The isotopic differences are attributed to differences in rates of excretion between the tissues and to differences in the isotopic signature of past versus present lead sources in the water. The lead isotopic compositions within the tissues reflect the degree of mixing of lead from the two different sources. Therefore, one of the reasons for the differences in lead isotopic abundances in the various tissues might be the combining lead from different sources.

In this study, the lead concentration in the diet, water and air is 139.27ng/g, 2.37ng/mL, and 2.16ng/L, respectively. According to adult rats' daily diet, water intake and alveolar ventilation, the rats (250g) in the low-dose group received 5 µg lead through intratracheal administration each day and about 1.74 µg, 0.06 µg and 0.12 µg lead intake through the diet, water and air, respectively. Compared to the lead acetate from intratracheal administrated, the lead from water and air are insignificant. Therefore, the intratracheal administrated lead acetate is the principal source of lead exposure in experimental groups; a smaller portion of lead comes from the diet. In addition, according to the previous study [12] and the figures, the lead isotopic compositions of tissues may not always follow a linear mixing line between the test substance and the diet. It may be speculated that the different enrichment or depletion capacity for each lead isotope among the diverse tissues might be another possible reason for the differences in Pb isotopic abundances in rats tissues.

In a lead distribution approach, the utilization rates (percentage) of lead isotopes in kidneys and liver significantly differed from that of other tissues in the rats fed with enriched stable isotope ^206^Pb[11]. Accordingly, the ^206^Pb/^208^Pb ratio of kidneys and liver is significantly discriminated. The results indicated that the difference in isotopic composition among tissues of rats may be related to the variation in the utilization rates of ^206^Pb. Thus, the variation in rates of utilization and excretion of exogenous lead isotopes may lead to differences in enrichment or depletion capacity for lead isotope among diverse tissues. However, the biological mechanisms that enrich or deplete a particular isotope for a given tissues is still not clear. Further studies are required to investigate this hypothesis.

It can be seen that the rank trend of lead isotope abundances in biological samples changed, when the rats received high relatively lead exposure. In addition, the lead isotope abundances of most biological sample pairs differed from each other in the control and low-dose group, while in medium- and high-dose groups the isotopic abundances of that became too close to discriminate. The findings suggest that the administrated dose maybe have an impact on the lead isotopic redistribution in different biological samples.

The whole-blood lead level in rats usually increases with the increasing dose of lead. Our data showed that the ^206^Pb% in liver and ^207^Pb% in bone were negatively associated with whole-blood lead levels, while the ^207^Pb% in liver was positively associated with whole-blood lead levels. Generally, when the whole-blood lead level exceeded the specific concentration, the differences in isotopic abundances between the tissue and the test substance became significantly smaller (Fig. 4B) or larger (Fig. 4A). No matter if the differences became smaller or larger; the variation in lead isotope abundances among biological samples always significantly decreased when the lead level exceeded the inflexion. This specific concentration of the whole-blood level is ∼ 50 ng/mL, which is consistent with our previous study. This phenomenon confirms the concept of the ‘fractionation functional threshold'. When the blood lead level exceeds this threshold, the biological fractionation function of the tissues is damaged and the lead isotope abundances of all biological samples tend to be constant.

According to the lead metabolism model, the blood is the central pool which is in direct communication with ingested lead, urinary lead, and pool two (soft tissue) and three (bone) [13]. The lead isotopes in the blood nearly have direct exchanges with that in most of lead stores. Thus, it is possible that the whole-blood level is related to the lead isotopic fingerprint of diverse tissues in rats. Any lead isotopic study that tries to accurately identify lead sources from the complex environment should consider the effect of the blood lead level on the biological fractionation function. Of course, the choice of biological samples should also be taken into account. At low blood lead level, the lead isotopic composition of samples from lead poisoned SD rats were statistically distinguishable, the biological sample that matches the lead sources well in lead isotopic composition should be chosen. According to our results, feces sample is more suitable for tracing lead sources at low blood lead level. At high blood lead level, because of the similar lead isotopic composition of different tissues, the choice of biological samples will not have critical effect on the identifying lead sources.

## Conclusions

Significant differences in lead isotope abundances are observed among kidneys, liver, lungs, bone, blood, urine and feces from the lead poisoned SD rats. The results confirm that the biological fractionation of lead isotopes occur in the biological system. The ^206^Pb,^ 207^Pb and ^208^Pb abundances in diverse samples show different degrees and directions of departure from the lead sources. In general, blood and urine samples have relatively strong capability of fractionation in lead isotopes. Based on the literatures and our results, it can be assumed that the different enrichment or depletion capacity for lead isotope among tissues might be one of the possible reasons for the variation in lead isotopic abundances in SD rats tissues. In addition, our data support the speculation of the fractionation functional threshold. When the blood lead level is higher than the threshold, the function of biological fractionation in lead isotopes is damaged and the lead isotopic composition in biological samples tends to be the same. Therefore, the blood lead level and the choice of biological samples should be taken in account when attempting to accurately identify lead sources from the complex environment by using lead isotope analysis.
